# HSPA6, a novel prognostic and therapeutic biomarker, associated with Ming classification in gastric cancer

**DOI:** 10.1002/jcla.24763

**Published:** 2022-12-01

**Authors:** Lihua Zhang, Hui‐qin Zhuo, Zhi‐jun Hong, Jing‐jing Hou, Jia Cheng, Jianchun Cai

**Affiliations:** ^1^ Department of Gastrointestinal Surgery Zhongshan Hospital of Xiamen University, School of Medicine, Xiamen University Xiamen China; ^2^ Institute of Gastrointestinal Oncology, School of Medicine Xiamen University Xiamen China; ^3^ Xiamen Municipal Key Laboratory of Gastrointestinal Oncology Xiamen China

**Keywords:** biomarker, gastric cancer, Hippo pathway, HSPA6, prognosis, proliferation

## Abstract

**Objective:**

This study aimed to explore the clinical relevance of heat shock protein family A member 6 (HSPA6) in gastric cancer (GC) and its effect on GC cell proliferation.

**Methods:**

HSPA6 mRNA and protein levels were analyzed by bioinformatics, RT‐qPCR, western blot and immunohistochemistry. HSPA6 was correlated with clinicopathological variables by the Chi‐square test. Kaplan–Meier survival analysis and the univariate and multivariate Cox models were used to assess the prognostic value of HSPA6. Nomogram was used to predict overall survival in patients with GC. Knockdown or over‐expression of HSPA6 in GC cell lines was constructed by lentiviral transduction. EdU and CCK‐8 assay were used to detect cell proliferation. In vivo mouse tumor models were performed to evaluate the effects of HSPA6 on GC growth.

**Results:**

HSPA6 were significantly upregulated in the GC tissues compared to the normal stomach epithelium and were associated with Ming classification (*p* < 0.001) and tumor size (*p* = 0.002). Patients with high expression of HSPA6 showed worse survival compared to the low expression group. HSPA6 was identified to be an independent prognostic biomarker for GC. HSPA6 was functionally annotated with the cell cycle, G2M checkpoint and Hippo pathway. Knockdown of HSPA6 suppressed XGC‐1 cell proliferation both in vitro and in vivo. Overexpression of HSPA6 in AGS cells increased proliferation rates, increased the levels of cyclinB1 and YAP and decreased that of phosphorylated YAP. HSPA6 knockdown in the NUGC2 cells had the opposite effect.

**Conclusions:**

HSPA6 promotes GC proliferation by the Hippo pathway, as a novel prognostic biomarker and potential therapeutic target.

## Graphical abstract

HSPA6 is up‐regulated in GC (a) The expression of HSPA6 in different cancers from Oncomine database. (b‐d) The expression of HSPA6 in STAD from (b) Oncomine database, (c) UALCAN and (d) GEPIA. (e) HSPA6 mRNA levels in STAD and normal tissues. (f) Immunoblot showing expression of HSPA6 protein in STAD and normal tissues and (g) the relative expression levels.

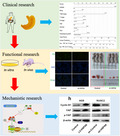

## 1 INTRODUCTION

Gastric cancer (GC) is also named stomach cancer, which ranked the fourth most commonly diagnosed malignancy and third in terms of mortality in the world.^1^ It is divided into infiltrative GC (IGC) and expanding GC (EGC) according to Ming classification, which is related to Bormann classification (protrusion and ulcer type), Lauren classification (intestinal and diffuse type) and WHO classification (papillary adenocarcinoma, adenosquamous carcinoma, squamous cell carcinoma, carcinoid, etc.).^2^ The incidence of IGC is 61.5% and its prognosis is worse compared to EGC.^3^ Despite considerable advances in molecular diagnosis and immunotherapy,^4^, ^5^ the mechanisms of the genesis and progression in GC remain unclear. In recent years several genes and non‐coding RNAs playing a vital role in GC development have been identified.^6^ The heat shock protein family A member 6 (HSPA6) gene is located on chr1:161,524,540‐161,526,894(GRCh38/hg38).^7^ HSPA6 is an autosomal recessive candidate protein with the function of the VATER/VACTERL malformation spectrum.^8^ Overexpression of HSPA6 inhibited the growth, migration and invasion of cells in triple‐negative breast cancer.^9^ HSPA6 exhibits to inhibit tumorigenesis and tumor progression in some types of cancers but promotes in others.^10^ Even though HSPA6 research has increased, its exact roles and mechanisms in GC are still unclear. Here, we aim to investigate the clinical relevance of HSPA6 and its biological role in GC. Firstly, we used RT‐qPCR, western blot and immunohistochemistry (IHC) methods to detect HSPA6 expression in GC. Secondly, we used the Chi‐square test to evaluate its correlation with clinical relevance and Kaplan–Meier method to evaluate its association with overall survival (OS). To demonstrate the clinical value of HSPA6 in GC, nomogram including HSPA6 was used to predict OS in patients with GC. Thirdly, to further explore the function of HSPA6 in GC, we performed an integrated transcriptomic analysis and predicted the molecular functions through bioinformatics. At last, HSPA6 was knocked down or overexpressed in GC lines to explore its biological function. The effects of HSPA6 on GC proliferation were performed by EdU assay in vitro and mouse tumor models in vivo. In the mechanism of HSPA6, cyclinB1 and YAP were detected by western blot. In all, this study would reveal HSPA6 is a novel prognostic biomarker and promotes tumor progression in GC.

## 2 MATERIALS AND METHODS

### 2.1 Patients and tissue samples

A total of 146 paired GC and normal stomach epithelial tissue samples were collected from patients underwent surgical resection at the Jiangnan University Hospital and Zhongshan Hospital of Xiamen University between March 2015 and January 2017. Freshly resected tissue from 20 patients were snap‐frozen in liquid nitrogen and other specimens from 146 patients were fixed in formalin and embedded in paraffin. This study was approved by the Ethics Committee of Jiangnan University Hospital and Zhongshan Hospital of Xiamen University. All patients involved in this study provided informed consent. The patients who received chemotherapy or radiotherapy before surgery were excluded. The mean time of follow‐up duration ranged from 0.12 to 7.3 years was 4.6 years. According to the guidelines of the American Joint Committee on Cancer (AJCC), the classification of GC tumor stage was determined by more than two pathologists who were blinded to the patients' data.

### 2.2 Bioinformatics analysis

The HSPA6 expression in mRNA levels from the GC and normal stomach epithelial tissue samples were analyzed by the integrated analysis of Coexpedia (www.coexpedia.org),^11^ Oncomine (www.oncomine.org)^12^ and UALCAN (http://ualcan.path.uab.edu/index.html)^13^ databases. The entire transcriptome data of 443 human GC tissue samples were downloaded from TCGA‐STAD datasets (https://www.cancer.gov/) and that of human‐derived GC cell lines from CCLE datasets (https://portals.broadinstitute.org/ccle). The HSPA6‐associated pathways and gene clusters were identified by Gene set enrichment analysis (GSEA) version 3.0 based on hallmark gene sets from the Molecular Signatures Database v7.4. Furthermore, enrichment analysis was performed by false discovery rate < 0.05 as the criteria for significantly enriched genes with a random combination number of 1000.^14^ The gene sets of HSPA6^hi^ and HSPA6^low^ groups were classified based on the median expression of HSPA6.

### 2.3 Cell culture

XGC‐1 had been successfully established from GC patients diagnosed with IGC by Ming classification.^15^ The XGC‐1, AGS and NUGC2 cell lines (Cell Bank of the Chinese Academy of Sciences) were cultured in RPMI‐1640 (Thermo Scientific) supplemented with 10% fetal bovine serum (Thermo Scientific) at 37°C with 5% CO_2_ and 90%–95% humidity.

### 2.4 RNA isolation and RT‐qPCR

Total RNA was extracted by Trizol reagent (Invitrogen) according to the manufacturer's instructions, and reversely transcribed by the Prime Script RT‐PCR kit (Takara). The cDNA template was amplified by the Prime Script RT‐qPCR on an ABI 7500 RT‐PCR (Applied Biosystems) with SYBR Green Master Mix (Takara), and normalized to β‐actin levels. The primers were as follows: HSPA6 forward 5′‐GATGTGTCGGTTCTCTCCATTG‐3′ and reverse 5′‐CTTCCATGAAGTGGTTCACGA‐3′, β‐actin forward 5′‐CCTGTGGCATCCACGAAACT‐3′ and reverse 5′‐GAAGCATTTGCG GTGGACGAT‐3′. All reactions were performed three times.

### 2.5 Western blotting

Total protein extracted by RIPA lysis buffer (Thermo Scientific) was determined with an enhanced bicinchoninic acid assay kit (CWBio). The protein from each sample was separated by sodium dodecyl sulfate‐polyacrylamide gel electrophoresis and transferred to polyvinylidene difluoride membranes (CWBio). The membranes blocked with 5% non‐fat milk for 1 h at room temperature (RT) later were incubated the whole night with primary antibodies targeting HSPA6 (1:2000, ab212044, Abcam), Cyclin B1 (1:3000, ab32053, Abcam), YAP1 (1:5000, ab56701, Abcam), p‐S127‐YAP1 (1:3000, ab226760, Abcam) and β‐actin (1:5000, ab8226, Abcam) at 4°C. The membranes were washed three times with tris‐buffered saline with Tween 60 (TBST) and probed with horseradish peroxidase‐conjugated secondary antibody (1:5000) for 1.5 h at RT. Protein bands were exposed by a chemiluminescence system, and the membranes were visualized by X‐ray films (Bio‐Rad). Densitometric analysis was performed by image software (Media Cybernetics). The relative protein expression levels of proteins measured in this study were normalized to β‐actin.

### 2.6 Immunohistochemistry

Tissue specimens used in this study were fixed, dehydrated, embedded and then cut into 5 μm‐thick sections. IHC was performed using a kit as per the manufacturer's protocol. Following antigen retrieval in hot citrate buffer, those sections were incubated the whole night with anti‐HSPA6 primary antibody (1:200, ab212044, Abcam) at 4°C. Each section of those was washed three times with TBST for 5 min, and incubated with a rabbit secondary antibody at RT for 1 h. The staining intensity of HSPA6 and the percentage of positively stained cells in tissues were scored by three independent pathologists in a blinded fashion. Based on the percentage of positively stained cells, the samples were graded as 4 (>76%), 3 (51%–75%), 2 (26%–50%), 1 (6%–25%) and 0 (≤5%). The staining intensity was graded as strong, 3 moderate, 2 weak 1 and negative (0). The total scores were calculated by multiplying the staining intensity score with that of the percentage of positive cells and ranged from 0 to 12.^16^ Subsequently, the samples were stratified as HSPA6^high^ and HSPA6^low^ based on scores ranging from 6 to 12 and 0 to 4, respectively.

### 2.7 Lentiviral transduction

The XGC‐1, AGS and NUGC2 cells were plated in dishes with 80% confluence 24 h prior to transfection. HSPA6 and control lentiviral vectors were transfected into the AGS cells, and HSAP6‐shRNA (GenePharma, Shanghai, China) into XGC‐1 and NUGC2 cells. The stably transfected cells were selected with 5 μg/mL puromycin, and HSAP6 expression was verified by RT‐qPCR and Western blotting.

### 2.8 EdU assay

5‐Ethynyl‐20‐deoxyuridine (EdU) cell proliferation assay was conducted to detect the proliferation of cell lines. According to EdU kit instruction (Beyotimes, China), HSPA6‐shRNA‐XGC‐1 cells and vector‐shRNA‐XGC‐1 cells were inoculated into a well from 24‐well plate at a density of 1 × 10^*5*^ cells and cultured for 6 h, then incubated with 200 μl of 100 μl EdU solution for 2 h, fixed in 4% formaldehyde, photographed under a Zeiss fluorescence microscope (Zeiss).

### 2.9 Cell Counting Kit‐8 (CCK‐8) assay

The proliferation rates of cells were detected by the CCK‐8 (Dojindo Laboratories). The suitably treated cells were seeded into 96‐well plates at the density of 2 × 10^3^ cells per well, and 10 μl CCK‐8 solution was added at the 0, 24, 48, 72, 96 and 120 h time points. After incubating for 2 h, the absorbance at 450 nm was recorded by a microplate reader (BioTek).

### 2.10 Mouse tumor models assay

The animal study and the experimental protocol were approved by the Xiamen University Experimental Animal Care Commission. BALB/c nude mice were purchased from the Animal Research Center of Xiamen University and fed according to institutional policies. Vector or HSPA6‐shRNA transfected XGC‐1 cells were collected in PBS, then injected into the mice for subcutaneous xenograft tumor models (5 × 10^6^ cells/mouse, *n* = 3/group). The animals were examined every 3 days for the development of tumors.

### 2.11 Statistical analysis

The performed statistical software was an R software (version 3.5.3). Student's t test was used to compare differences in HSPA6 expression between tumor and normal samples. The associations between HSPA6 and the clinicopathological features in GC were assessed by Fisher's exact test or Chi‐square test when appropriate. A nomogram was constructed based on the results of the multivariate analysis with rms package in R version 3.5.3 (http://www.r‐project.org/). The OS curve was plotted using the Kaplan–Meier. The univariate and multivariate analyses containing factors of TNM stage, HSPA6 and Ming classification were performed by Cox proportional hazard regression model. All *P* values were two‐tailed and considered statistically significant as *p <* 0.05.

## 3 RESULTS

### 3.1 HSPA6 is overexpressed in GC tissues

**FIGURE 1:**
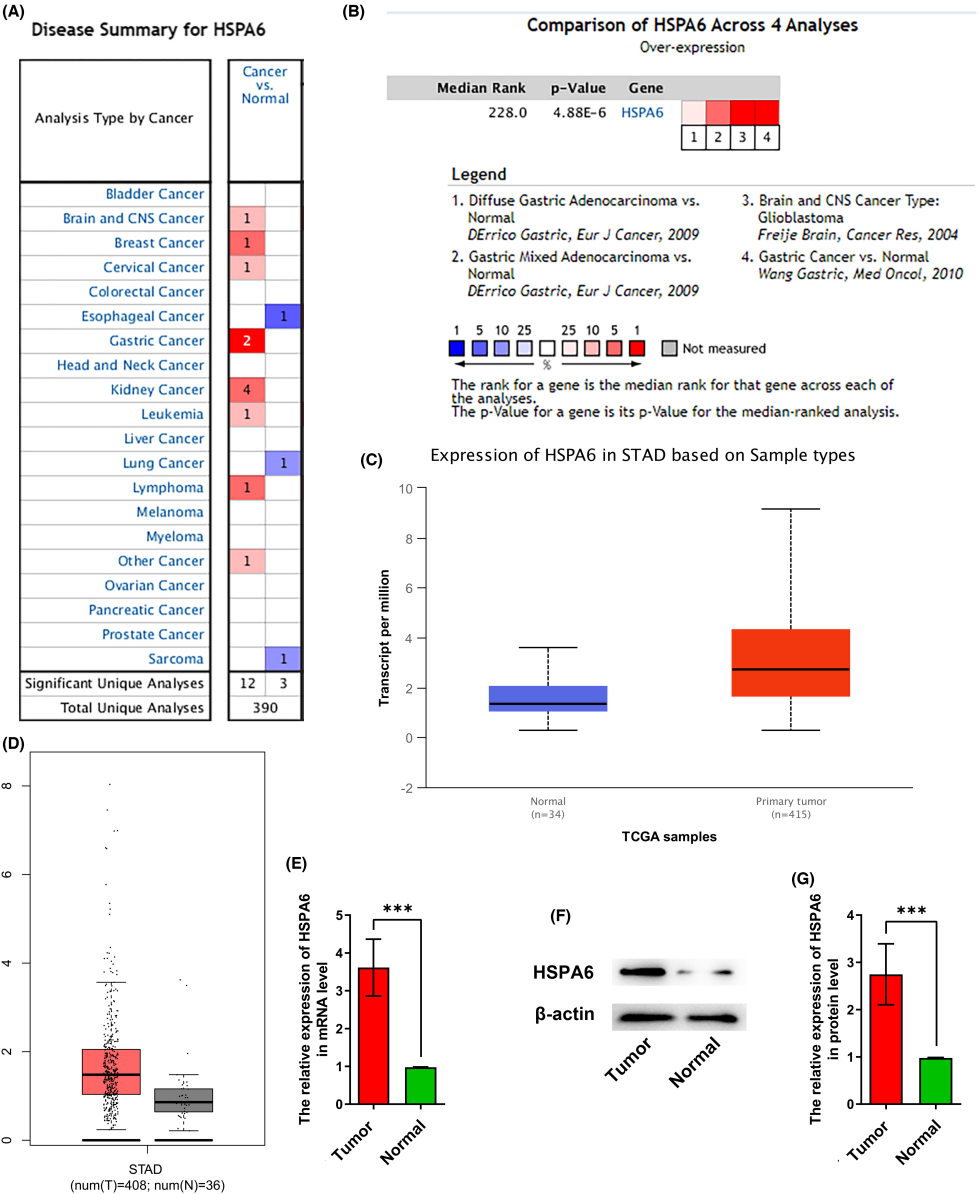
HSPA6 is up‐regulated in GC. (A) The expression of HSPA6 in different cancers from the Oncomine database. (B–D) The expression of HSPA6 in STAD from (B) Oncomine database, (C) UALCAN and (D) GEPIA. (E) HSPA6 mRNA levels in STAD and normal tissues. (F) Immunoblot showing expression of HSPA6 protein in STAD and normal tissues and (G) the relative expression levels.

Screening of the Oncomine database revealed that HSPA6 is overexpressed in kidney cancer, brain and CNS cancers, leukemia and lymphoma, breast cancer, cervical cancer, colorectal cancer and GC (Figure 1A). In addition, the significantly higher expression levels of HSPA6 in GC tissues were also confirmed through previously published studies (Figure 1B) as well as the GEPIA and UALCAN databases (Figure 1C,D). Consistent with previous reports, we found that the HSPA6 expression in mRNA level was significantly up‐regulated in the GC tissues compared to the normal gastric epithelial tissues (Figure 1E). The HSPA6 protein was also significantly up‐regulated in the GC tissues compared with the normal gastric epithelial tissues (Figure 1F,G). These findings suggest that HSPA6 is a potential oncogene in GC.

### 3.2 HSPA6 overexpression is related to poor prognosis and IGC

**FIGURE 2:**
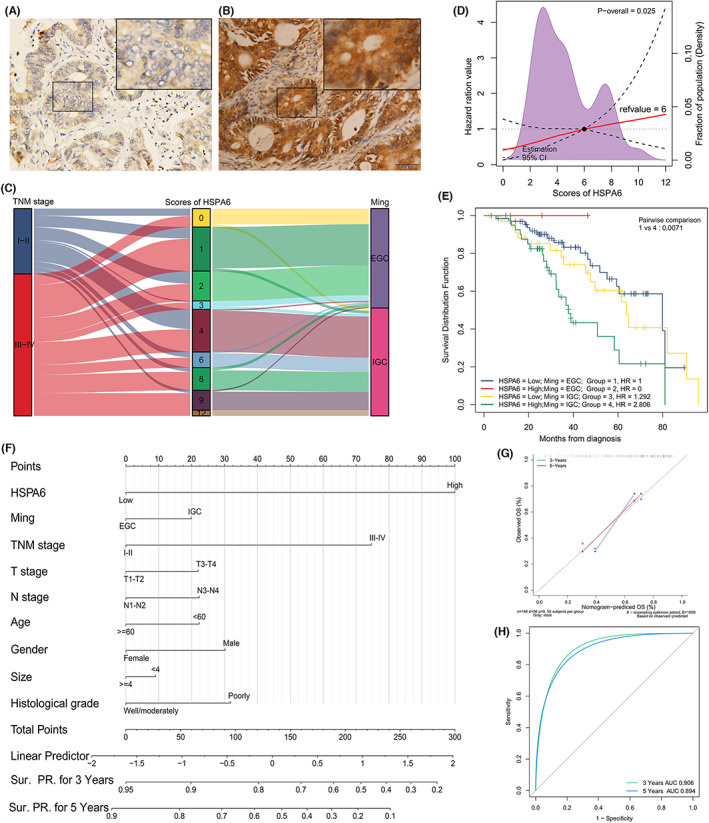
HSPA6 overexpression predicts poor survival in GC. (A, B) Representative images showing (A) low and (B) high in situ expression of HSPA6 in GC tissues; (C) Sankey diagram of TNM stage, HSPA6 expression and Ming classification; (D) restricted cubic splines for best cutoff point; (E) Kaplan–Meier overall survival curves of HSPA6^high^ and HSPA^low^ in EGC and IGC groups; (F) prognostic nomogram for GC based on HSPA6 expression and clinicopathological variables; (G, H) straight line graph (G) and time‐dependent receiver operating characteristic (ROC) curves (H) for evaluating the predictive power of the nomogram.

Based on the IHC staining score of HSPA6 (Figure 2A,B), the GC samples were stratified into the HSPA6^high^ and HSPA6^low^ groups by the best cutoff score point at 6 scores (Figure 2D). The expression of HSPA6 was significantly correlated with tumor size (*p =* 0.002, Table 1) and Ming classification (*p* < 0.001, Table 1) but not with age, gender, tissue grading and N‐phases (*p* > 0.05, Table 1). The correlation of HSPA6 expression with Ming classification was intuitively displayed by Sankey diagram (Figure 2C). HSPA6^high^ in the IGC group showed more significantly poor survival rates compared to that in HSPA6^low^ in the EGC group (*p* < 0.01, Figure 2E). In addition, univariate and multivariate COX analyses identified TNM stage and HSPA6 as independent prognostic factors of GC (*p* < 0.05, Table 2). The nomogram model for the prediction of 3 or 5 years survival rate was developed based on multivariate logistic regression (Figure 2F). The goodness‐of‐fit of the nomogram model was evaluated by the A Hosmer–Lemeshow test and was ideal (Figure 2G). The area under the ROC curve (AUC) was used to measure the discrimination performance. The AUC for 3 and 5 years were 0.906 and 0.894, respectively (Figure 2H). Those results indicated that the model was a good performance.

**TABLE 1:** The correlation between HSPA6 expression and clinicopathological variables in GC.

Characteristics	No. cases	HSPA6 expression		χ^2^	*p* Value
		Low	High		
Age					
<=60	83	56	27	0.11	0.739
>60	63	45	18		
Gender					
Female	84	56	28	0.34	0.559
Male	62	45	17		
T stage					
T1–T2	73	55	18	2.05	0.152
T3–T4	73	46	27		
N stage					
N0	64	40	24	1.86	0.173
N1–N3	82	61	21		
TNM stage					
I–II	46	37	9	3.25	0.071
III–IV	100	64	36		
Histological grade					
Poor and middle	73	47	26	1.15	0.282
Well	73	54	19		
Tumor size					
<=4 cm	65	54	11	**9.47**	**0.002**
>4 cm	81	47	34		
Ming classification					
EGC	70	66	4	**37.53**	**<0.001**
IGC	76	35	41		

Bold face indicates statistical significance.

**TABLE 2:** The prognostic factors of GC through univariate and multivariate COX regression analysis.

Characteristics	Univariate analysis		Multivariate analysis	
	HR (95%CI)	*p* value	HR (95%CI)	*p* value
Age (>60 vs ≤60)	0.784(0.531–1.155)	0.218		
Gender (male vs female)	1.29(0.886–1.879)	0.184		
Size (>4 cm vs <4 cm)	1.173(0.794–1.733)	0.424		
Histological grade (poorly vs well/moderately)	1.141(0.776–1.677)	0.502		
T stage (T3–T4 vs.T1–T2)	0.746(0.511–1.09)	0.130		
N stage (N2–N3 vs.N0–N1)	0.919(0.631–1.337)	0.658		
TNM stage (III–IV vs.I‐II)	**1.654(1.124–2.434)**	**0.011**	**1.999(1.323–3.02)**	**<0.001**
HSPA6 (high vs low expression)	**2.28(1.34–3.86)**	**0.002**	**2.23(1.31–3.78)**	**0.003**
Ming classification (IGC vs EGC)	**1.596(1.067–2.385)**	**0.023**	1.198(0.749–1.915)	0.450

Bold face indicates statistical significance.

### 3.3 Knocking down HSPA6 suppressed GC proliferation both in vitro and in vivo

**FIGURE 3:**
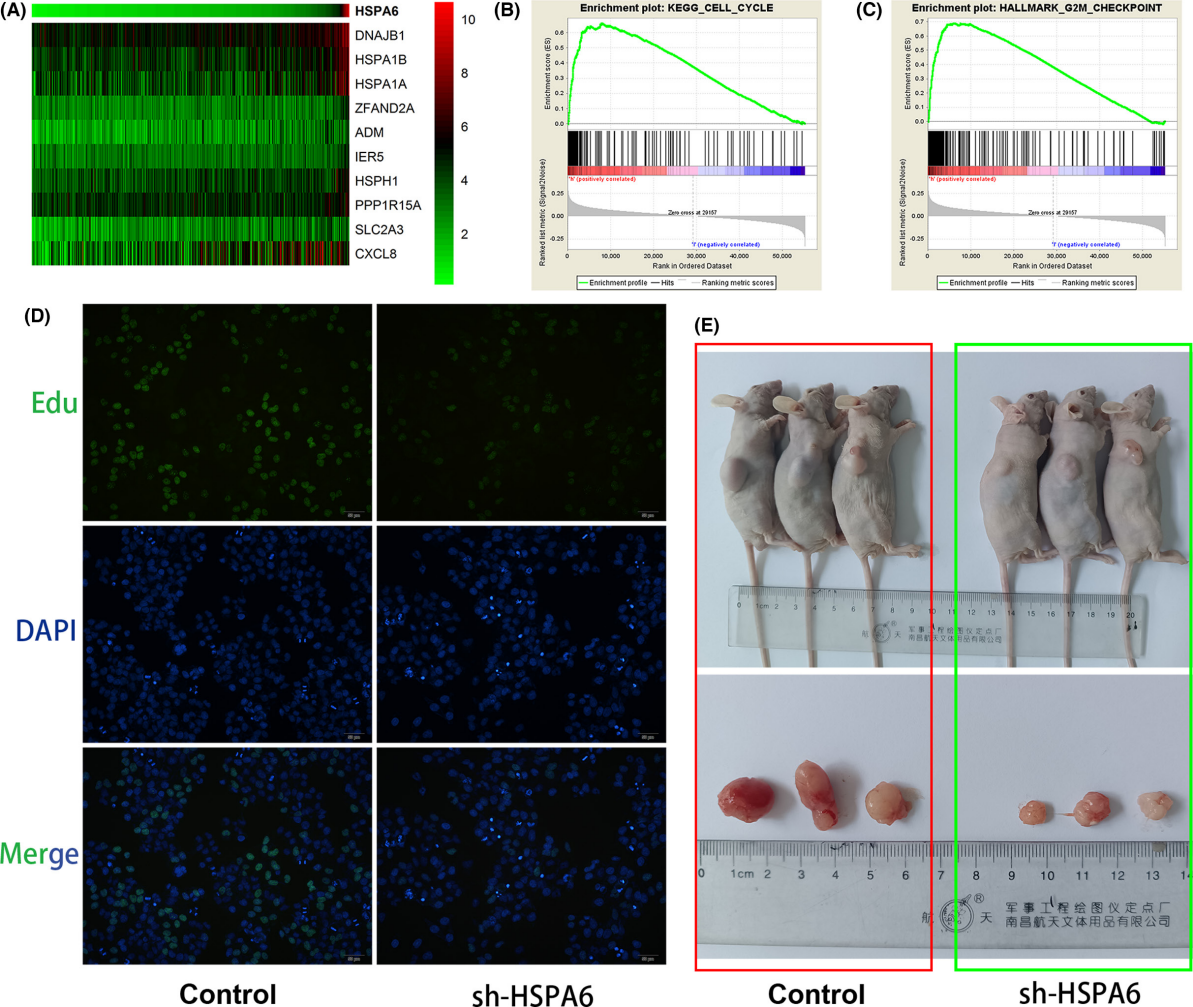
HSPA6 is associated with the proliferation of GC. (A) Heatmap showing the top 10 genes correlated to HSPA6 in the TCGA‐STAD dataset. (B) The significantly enriched KEGG pathways of HSPA6‐related genes. (C) The significantly enriched Hallmark functions of HSPA6‐related genes. (D) The proliferation of cells between the control and sh‐HSPA6 group was measured by 5‐ethynyl‐20‐deoxyuridine (EdU). (E) Representative images of tumor tissues in each group were shown.

**FIGURE 4:**
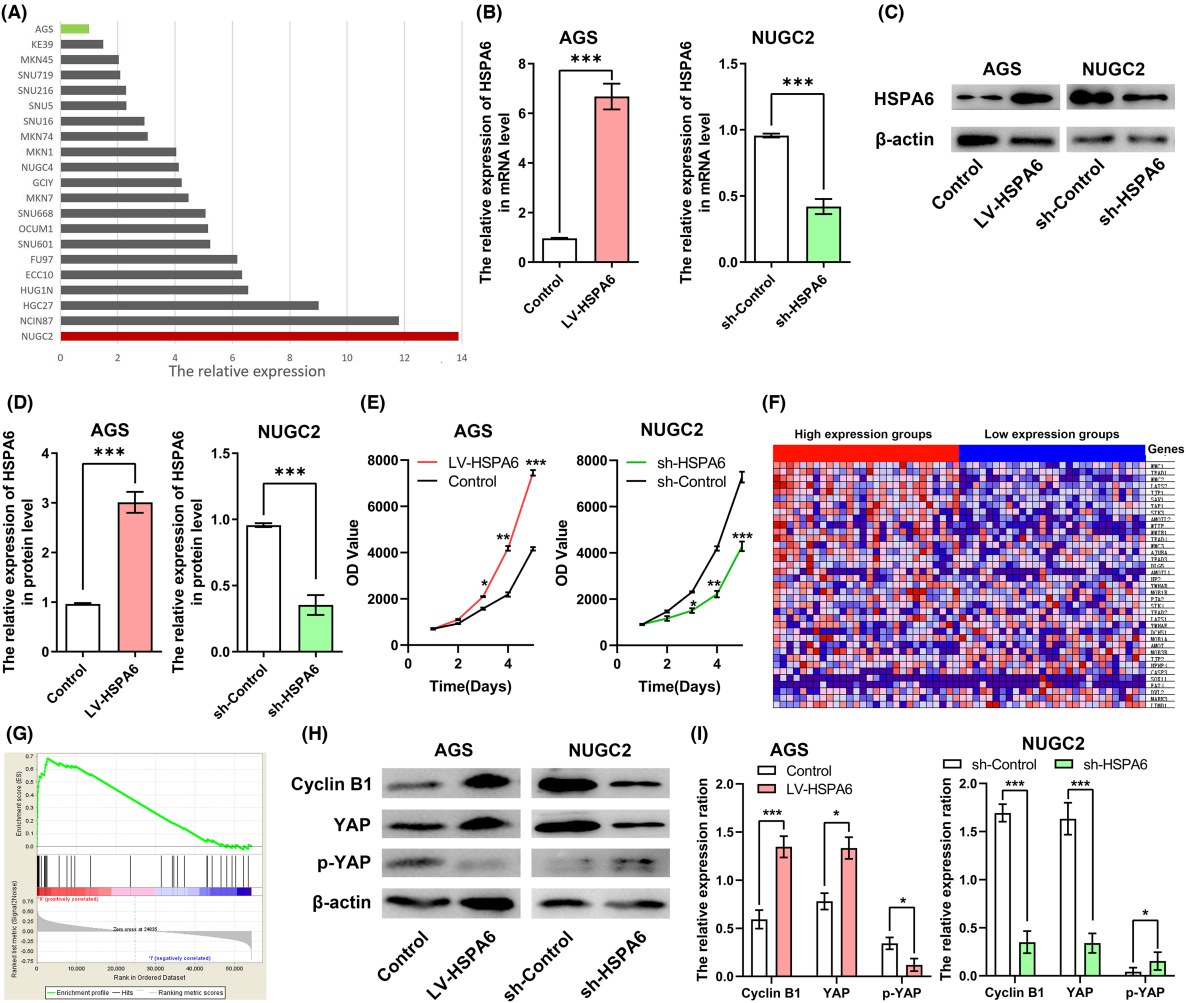
HSPA6 is up‐regulated in GC. (A) The expression of HSPA6 in STAD cell lines from the CCLE database. (B) RT‐qPCR results showing HSPA6 mRNA levels in AGS and NUGC2 cells respectively transfected with HSPA6 overexpression and siRNA constructs. (C) Immunoblot showing HSPA6 protein in the indicated cell lines and (D) the relative expression levels. (E) The growth curve of the indicated cell lines. (F) Heatmap showing the top 20 genes correlated with HSPA6 expression in STAD cell lines from CCLE. (G) The significantly enriched KEGG pathways of HSPA6‐related genes. (H) Immunoblots showing cyclin B1, YAP and p‐YAP proteins in the indicated cell lines and (I) their relative expression levels.

To further assess the molecular function of HSPA6 in GC, we analyzed the gene set associated with HSPA6 using the STAD transcriptome data from the TCGA database. The top 10 genes correlated with HSPA6 in STAD are outlined in the heatmap (Figure 3A). Furthermore, the results from GSEA showed that the significantly enriched KEGG pathway and hallmark function of these genes were cell cycle (Figure 3B) and G2M checkpoint (Figure 3C) respectively, indicating that HSPA6 is relevant to the proliferation of GC cells. In vitro assay, Edu incorporation was markedly lower in the HSPA6‐knockdown XGC‐1 cells relative to the controls (Figure 4D). Consistent with this in vivo assay, mouse tumor models showed that the weight of the HSPA6‐knockdown XGC‐1 cells (0.40 ± 0.28 g) was significantly lower than that of the respective controls (1.72 ± 0.77 g, Figure 4E).

### 3.4 HSPA6 promotes GC proliferation via activating the Hippo pathway

The transcriptome data of multiple GC cell lines were downloaded from CCLE and showed that HSPA6 was downregulated in AGS cells and highly expressed in NUGC2 cells (Figure 4A). To experimentally determine the biological role of HSPA6 in GC, we overexpressed HSPA6 in the AGS cells and inactivated it in the NUGC2 cells using siRNA (Figure 4B–D). Overexpression of HSPA6 significantly increased the proliferation of AGS cells compared to the control group, while HSPA6 silencing had the opposite effect in the NUGC2 cells (Figure 4E), thereby confirming the bioinformatics findings. Furthermore, the genes associated with high and low HSPA6 expression were also identified from the transcriptome data of GC cell lines. The top 20 genes are shown in the heatmap in Figure 4F. GSEA revealed that the Hippo signaling pathway was significantly enriched in the HSPA6‐related genes, indicating that HSPA6 might promote the proliferation of GC cells through the Hippo signaling pathway (Figure 4G). Therefore, we detected the expression levels of cyclin B1, YAP and p‐YAP proteins in the AGS and NUGC2 cells. Overexpression of HSPA6 in the AGS cells significantly increased the expressions of cyclin B1 and YAP, and decreased that of p‐YAP. Knocking down HSPA6 in the NUGC2 cells led to opposite changes (Figure 4H,I). Taken together, HSPA6 promotes the proliferation of GC cells by activating the Hippo pathway.

## 4 DISCUSSION

Currently, GC is treated through surgery, radiotherapy and chemotherapy.^4^ In addition, the identification of novel molecular markers of GC, such as genes,^17^ miRNAs,^18^ lncRNAs^19^ and cirRNAs,^20^ in recent years has accelerated the development of precision therapies. HSPA6 belongs to the HSP70 family and appears evolutionary unique as it is not conserved in rodents. HSPA6 expression is not detectable in most cells under normal conditions.^10^ Recent studies have found that HSPA6 exhibits to inhibit tumorigenesis and tumor progression in some types of cancers but promotes it in others. However, HSPA6 expression and its exact roles in GC are still unclear. We found that HSPA6 is aberrantly expressed in the GC tissues compared to normal stomach epithelium through integrated transcriptome analysis as well as direct analysis of patient samples from GEPIA and UALCAN databases. Furthermore, demarcation of the patients on the basis of in situ HSPA6 expression indicated that overexpression of HSPA6 correlated significantly with larger tumors and IGC. The OS of the HSPA6^high^ group was also worse compared to that of HSPA6^low^ patients, indicating that high expression of HSPA6 in GC tissues portends a poor prognosis. The results of COX analyses confirmed that HSPA6 is an independent prognostic factor for GC. Finally, The nomogram was constructed HSPA6 and clinical stage with a high performance of prediction. Those results indicated HSPA6 is a potential prognostic marker as well as an oncogene.

To further determine the functional relevance of HSPA6 in GC, we used GSEA to identify the pathways enriched in the genes correlated with HSPA6 expression levels in GC from the TCGA database. The cell cycle, G2M checkpoint and Hippo pathway were significantly enriched, which suggested that HSPA6 likely promotes the proliferation of the GC cells by activating the Hippo pathway. Indeed, knocking down HSPA6 suppressed cell proliferation in vitro and tumor growth in vivo. What's more, ectopic expression of HSPA6 in the HSPA6^low^ AGS cell line significantly increased proliferation rates, whereas gene silencing in the HSPA6^high^ NUGC2 cells inhibited growth. Consistent with our findings, overexpression of HSPA6 in the bladder cancer^21^ and lung cancer^22^ cells upregulated cyclin B1 and YAP and decreased the levels of phosphorylated YAP (p‐YAP). Cyclin B1 drives cell cycle progression through the G2/M checkpoint,^23^ and its high levels in GC cells are associated with accelerated G2/M transition.^24^ The transcriptional coactivator YAP of the Hippo signaling pathway associated with cell proliferation and organ size is an established oncoprotein in GC.^25^, ^26^ Taken together, HSPA6 promotes the proliferation of GC cells via activating the Hippo pathway.

There are several limitations in this study that ought to be considered. First, the number of samples was limited and the study design was retrospective. Secondly, the mechanistic basis of HSPA6 action was also unclear. Therefore, our findings would be validated on a larger patients' cohort. In addition, pulldown, protein MS and Co‐IP experiments will be needed to clarify the molecular mechanism of HSPA6.

## 5 CONCLUSION

Our study demonstrates that HSPA6, a novel prognostic biomarker and promising therapeutic target for GC, is overexpressed in GC and promotes proliferation via the Hippo pathway.

## AUTHOR CONTRIBUTIONS

Cai JC designed the research; Zhang LH, Hou JJ, Hong ZJ and Zhuo HQ performed the research; Cai JC contributed new reagents/analytic tools; Zhang LH, Cheng J and Hong ZJ analyzed the data and Zhang LH and Zhuo HQ wrote the paper.

## FUNDING INFORMATION

This work was supported by National Natural Science Foundation of China (81871979); Natural Science Foundation of Fujian Province (2021J05276 and 2021J02056) and the Medical and Health Sciences Foundation of Xiamen (3502Z20199171 and 3502Z20204002).

## CONFLICT OF INTEREST

The authors report no conflicts of interest in this work.

## Data Availability

All data generated or analyzed during this study are included in this published article.

## References

[1] Sung H , Ferlay J , Siegel RL , et al. Global cancer statistics 2020: GLOBOCAN estimates of incidence and mortality worldwide for 36 cancers in 185 countries. CA Cancer J Clin. 2021;71(3):209‐249. 33538338 10.3322/caac.21660

[2] Luebke T , Baldus SE , Grass G , et al. Histological grading in gastric cancer by ming classification: correlation with histopathological subtypes, metastasis, and prognosis. World J Surg. 2005;29(11):1422‐1427. discussion 1428. 16222448 10.1007/s00268-005-7795-z

[3] Cheng J , Zhuo H , Wang L , et al. Identification of the combinatorial effect of miRNA family regulatory network in different growth patterns of GC. Mol Ther Oncolytics. 2020;17:531‐546. 32637572 10.1016/j.omto.2020.03.012 PMC7321821

[4] Joshi SS , Badgwell BD . Current treatment and recent progress in gastric cancer. CA Cancer J Clin. 2021;71(3):264‐279. 33592120 10.3322/caac.21657 PMC9927927

[5] Kwon M , An M , Klempner SJ , et al. Determinants of response and intrinsic resistance to PD‐1 blockade in microsatellite instability‐high gastric cancer. Cancer Discov. 2021;11:2168‐2185. 33846173 10.1158/2159-8290.CD-21-0219

[6] Ray K . New markers and models of premalignancy and the early development of gastric cancer. Nat Rev Gastroenterol Hepatol. 2020;17(4):193. 32103202 10.1038/s41575-020-0280-1

[7] Leung TK , Hall C , Rajendran M , Spurr NK , Lim L . The human heat‐shock genes HSPA6 and HSPA7 are both expressed and localize to chromosome 1. Genomics. 1992;12(1):74‐79. 1346391 10.1016/0888-7543(92)90409-l

[8] Kause F , Zhang R , Ludwig M , et al. HSPA6: a new autosomal recessive candidate gene for the VATER/VACTERL malformation spectrum. Birth Defects Res. 2019;111(10):591‐597. 30887706 10.1002/bdr2.1493 PMC6662190

[9] Shen S , Wei C , Fu J . RNA‐sequencing reveals heat shock 70‐kDa protein 6 (HSPA6) as a novel thymoquinone‐upregulated gene that inhibits growth, migration, and invasion of triple‐negative breast cancer cells. Front Oncol. 2021;11:667995. 34017687 10.3389/fonc.2021.667995 PMC8129564

[10] Song B , Shen S , Fu S , Fu J . HSPA6 and its role in cancers and other diseases. Mol Biol Rep. 2022;49:10565‐10577. 35666422 10.1007/s11033-022-07641-5

[11] Yang S , Kim CY , Hwang S , et al. COEXPEDIA: exploring biomedical hypotheses via co‐expressions associated with medical subject headings (MeSH). Nucleic Acids Res. 2017;45(D1):D389‐D396. 27679477 10.1093/nar/gkw868 PMC5210615

[12] Rhodes DR , Kalyana‐Sundaram S , Mahavisno V , et al. Oncomine 3.0: genes, pathways, and networks in a collection of 18,000 cancer gene expression profiles. Neoplasia. 2007;9(2):166‐180. 17356713 10.1593/neo.07112 PMC1813932

[13] Chandrashekar DS , Bashel B , Balasubramanya SAH , et al. UALCAN: a portal for facilitating tumor subgroup gene expression and survival analyses. Neoplasia. 2017;19(8):649‐658. 28732212 10.1016/j.neo.2017.05.002 PMC5516091

[14] Zhang L , Li L , Mao Y , Hua D . VGLL3 is a prognostic biomarker and correlated with clinical pathologic features and immune infiltrates in stomach adenocarcinoma. Sci Rep. 2020;10(1):1355. 31992826 10.1038/s41598-020-58493-7 PMC6987121

[15] Peng J , Xu H , Cai J . Establishment and characterization of a new gastric cancer cell line, XGC‐1. Cancer Cell Int. 2020;20:437. 32943986 10.1186/s12935-020-01536-w PMC7487967

[16] Zhang LH , Wang Y , Fan QQ , et al. Up‐regulated Wnt1‐inducible signaling pathway protein 1 correlates with poor prognosis and drug resistance by reducing DNA repair in gastric cancer. World J Gastroenterol. 2019;25(38):5814‐5825. 31636474 10.3748/wjg.v25.i38.5814 PMC6801184

[17] Wang F , Wu X , Li Y , Cao X , Zhang C , Gao Y . PFKFB4 as a promising biomarker to predict a poor prognosis in patients with gastric cancer. Oncol Lett. 2021;21(4):296. 33732372 10.3892/ol.2021.12557 PMC7905623

[18] Zheng GD , Xu ZY , Hu C , et al. Exosomal miR‐590‐5p in serum as a biomarker for the diagnosis and prognosis of gastric cancer. Front Mol Biosci. 2021;8:636566. 33681295 10.3389/fmolb.2021.636566 PMC7928302

[19] Wang W , Wu J . Identification of long noncoding RNA TC0101441 as a novel biomarker for diagnosis and prognosis of gastric cancer. Int J Clin Exp Pathol. 2021;14(3):363‐368. 33786153 PMC7994149

[20] Ma S , Kong S , Gu X , et al. As a biomarker for gastric cancer, circPTPN22 regulates the progression of gastric cancer through the EMT pathway. Cancer Cell Int. 2021;21(1):44. 33430866 10.1186/s12935-020-01701-1 PMC7802183

[21] Shin SS , Song JH , Hwang B , et al. HSPA6 augments garlic extract‐induced inhibition of proliferation, migration, and invasion of bladder cancer EJ cells; implication for cell cycle dysregulation, signaling pathway alteration, and transcription factor‐associated MMP‐9 regulation. PLoS One. 2017;12(2):e0171860. 28187175 10.1371/journal.pone.0171860 PMC5302316

[22] Wang L , Hou J , Wang J , et al. Regulatory roles of HSPA6 in actinidia chinensis planch. root extract (acroots)‐inhibited lung cancer proliferation. Clin Transl Med. 2020;10(2):e46. 32508044 10.1002/ctm2.46 PMC7403824

[23] Yasuda M , Takesue F , Inutsuka S , Honda M , Nozoe T , Korenaga D . Overexpression of cyclin B1 in gastric cancer and its clinicopathological significance: an immunohistological study. J Cancer Res Clin Oncol. 2002;128(8):412‐416. 12200597 10.1007/s00432-002-0359-9 PMC12164493

[24] Ding L , Yang L , He Y , et al. CREPT/RPRD1B associates with aurora B to regulate cyclin B1 expression for accelerating the G2/M transition in gastric cancer. Cell Death Dis. 2018;9(12):1172. 30518842 10.1038/s41419-018-1211-8 PMC6281615

[25] Han X , Sun T , Hong J , et al. Nonreceptor tyrosine phosphatase 14 promotes proliferation and migration through regulating phosphorylation of YAP of hippo signaling pathway in gastric cancer cells. J Cell Biochem. 2019;120(10):17723‐17730. 31168824 10.1002/jcb.29038

[26] Wang W , Liu Z , Chen X , et al. Downregulation of FABP5 suppresses the proliferation and induces the apoptosis of gastric cancer cells through the hippo signaling pathway. DNA Cell Biol. 2021;40:1076‐1086. 34160301 10.1089/dna.2021.0370

